# The Role of Nanofluids in Renewable Energy Engineering

**DOI:** 10.3390/nano13192671

**Published:** 2023-09-29

**Authors:** Muhammad Mubashir Bhatti, Kambiz Vafai, Sara I. Abdelsalam

**Affiliations:** 1 College of Mathematics and Systems Science, Shandong University of Science and Technology, Qingdao 266590, China; 2 Department of Mechanical Engineering, University of California, Riverside, CA 92521, USA; vafai@engr.ucr.edu; 3 Basic Science, Faculty of Engineering, The British University in Egypt, Al-Shorouk City, Cairo 11837, Egypt; sara.abdelsalam@bue.edu.eg

The phenomenon of nanofluid flows is intrinsically characterized by several scales and intricate physical processes. Numerous modeling and experimental methodologies have been extensively used to examine nanofluids, including both basic and applied perspectives. Significant advancements have been achieved in the field of physical understanding and research tools, with more substantial contributions anticipated in the future.

In recent times, the field of thermal technology has encountered many obstacles, with the realization of an elevated heat transfer rate emerging as a significant concern. Renewable energy often fails to attain the elevated temperatures attained by burning conventional fuels. Nanofluids have been shown to provide advantages in addressing these challenges. The fundamental principles of enhancing heat transmission through the use of nanofluids have been widely accepted and acknowledged in the academic literature. Further research is necessary to explore crucial domains that pertain to the use of nanofluids in energy-related applications. The phenomenon under consideration encompasses the interactions between nanoparticles and the base fluid, as well as the following effects these interactions have on heat convection. Moreover, the extensive use of nanofluids in renewable energy technologies represents a significantly underexplored domain. Numerous investigations have been carried out pertaining to nanofluids, specifically emphasizing their utilization in solar thermal and geothermal technologies, heat storage and networks, and other heat recovery technologies. A persistent obstacle in these domains is directed toward creating cost-effective computational tools that possess both accuracy and the ability to forecast the heat transfer characteristics of nanofluids. Consequently, it is necessary to conduct more experimental investigations on nanofluids at a systemic level, namely in applications such as solar thermal collectors, geothermal boreholes, and other renewable energy systems of significant size.

The engineering research community is confronted with a significant undertaking in the form of decarbonizing energy by shifting from fossil fuels to renewable sources. Thermal energy represents a substantial portion of the energy utilized in various regions globally and has a significant role in releasing greenhouse gases. In recent times, several nations have made the strategic choice to undertake the process of decarbonizing their respective energy sectors. Consequently, there is a growing focus on carbon-free thermal technologies, including solar thermal, geothermal, heat pumps, heat storage, and heat networks. The core issue here is the difficulty associated with transmitting thermal energy.

The primary objective of this Special Issue is to consolidate the most recent research discoveries pertaining to heat transfer facilitated by nanofluids, with a particular focus on the challenges associated with renewable energy. Next, this Special Issue collected nine selected original research papers and three comprehensive reviews on various topics in nanotechnology. A total of 68 scientists hailing from various universities and research organizations actively contributed their research efforts and knowledge, therefore playing a significant role in completing this Special Issue. Below is a summary of their scientific contributions.

The study by Ampah et al. [1] provided a comprehensive overview of the advancements in the use of nanoparticles in low-carbon fuels. The investigators noted that Asia is the primary driver of advancements in the field of biofuels, namely in the areas of biodiesel, vegetable oils, and alcohols. Nevertheless, significant emphasis has been placed on biodiesel. The findings of their study indicate that the combustion of low-carbon fuel diesel blends was boosted by the presence of nanoparticles in diesel engines. Nanoparticles function as an oxygen reservoir, supplying supplementary oxygen molecules inside the combustion chamber. This mechanism serves to reduce unburnt emissions and enhance the combustion process. The exceptional catalytic characteristics of nanoparticles are attributed to their behavior.

Nabwey et al. [2] examined the latest developments in the domain of porous media flow, including heat transfer and nanofluids. The authors set their evaluation on a curated collection of papers published within the period spanning 2018 to 2020. The participants engaged in a comprehensive discussion on several analytical methodologies for analyzing nanofluid flow inside porous media. The initial emphasis of the study was on the heat transfer phenomenon of natural convection in porous media. Subsequently, the researchers proceeded to examine heat transfer under forced convection and mixed convection scenarios. Based on their investigation, it was observed that altering the height of the medium results in a modification of the flow regime inside the chamber.

The primary emphasis of Moghaieb et al. [3] was on the advancements of nanofluids for use in direct-absorption solar collectors (DASCs). Their research primarily examined the optical properties, solar thermal conversion efficiency, production methods, and thermal and physical stability of these nanofluids. The authors also emphasized the difficulties associated with the actual use of nanofluids in direct-absorption solar collectors (DASCs) and offered recommendations for further research.

Hu et al. [4] provided an overview of the latest advancements in the development of solar thermal nanofluids capable of achieving homogeneous and stable dispersion at moderate temperatures. The authors comprehensively examined the issues associated with dispersion in several media, including ionic liquid, oil, molten salt-based, medium-temperature solar thermal nanofluids, and ethylene glycol. They conducted an in-depth investigation of the dispersion mechanism regulating these media and proposed a representative method. The benefits and application of many methods for strengthening the dispersion stability of thermal storage nanofluids were explored. These methods include electrostatic stabilization, self-dispersion stabilization, hydrogen bonding, steric stabilization, etc. Enhancing the dispersion stability of nanofluids used in medium-temperature solar thermal systems has the potential to not only encourage further investigation into the efficient absorption of solar thermal energy but also provide a promising solution to address basic challenges and limitations associated with nanofluid technology.

Zubair et al. [5] used a combination formulation of integrated fractional calculus and plasma modeling to examine the characteristics of the higher dimensional time fractional Vlasov–Maxwell system. A numerical methodology was devised for filtered Gegenbauer polynomials and temporal variables, demonstrating robustness, efficiency, speed, and accuracy. This approach combined a finite difference method with spectral approximations. Two kinds of boundary conditions were used, namely partial slip and Dirichlet.

Qureshi et al. [6] examined hybrid nanofluids, including several kinds of nanoparticles, namely Al_2_O_3_, Ti_2_O, and Fe_3_O_4_. The authors investigated the morphological characteristics of these nanofluids and explored the influence of a magnetic field on their properties. The flow is presumed to be transitioning between two coaxially spinning disks. The numerical simulation used the Runge–Kutta technique in conjunction with the shooting method. It has been shown that there exists a direct relationship between the shape of nanoparticles and the heat conductivity of nanofluids.

Bhatti et al. [7] introduced a mathematical model that describes the flow of a nanofluid consisting of Magnesium oxide (MgO) and nickel (Ni) particles, taking into account the influence of magnetization. The researchers assumed a stagnation point flow occurring over an elastic porous surface. They employed homogeneous porous media with the assistance of Darcy law. The research also considers slip and thermal slip boundary conditions. They discovered that the thermal boundary layer thickness and thermal profile were greatly enhanced owing to the influence of magnesium oxide and nickel nanoparticles. The thermal profile was also found to be boosted owing to the heat production while it was suppressed due to the thermal slip effects. Furthermore, hybrid nanofluids have exhibited significant improvements in thermal enhancement. 

Rehman et al. [8] employed carbon nanotubes to investigate the characteristics of nanofluids. Their research revealed that carbon nanotubes offer distinct advantages over other types of nanofluids. The authors considered a time-dependent stagnation point flow occurring on an elastic surface. To analyze the problem, they utilized an optimized version of the homotopy analysis method. The outcomes of their investigation demonstrated that nanoparticles, the unsteady parameter, and the magnetic field exhibited an opposing effect on the velocity. Conversely, the Eckert and Prandtl numbers displayed varying influences on the thermal profile.

The properties of nanofluid flows, which have been constructed based on basic principles and experimental data, were described by Rizwan et al. [9]. Various types of nanoparticles, including SiO_2_, TiO_2_, and MgO, were included in the study using a power law model. The researchers used a numerical shooting approach to assess the obtained findings. An observed decrease in the velocity profile has been attributed to the increasing concentration of nanoparticles, whereas a concurrent rise in the thermal profile has been noted. Nevertheless, the size of the nanoparticles has an inverse effect on both the velocity and heat profile. The findings of this study have practical implications for a range of industrial applications that need a comprehensive understanding of heat and mass transfer phenomena to optimize device design.

The study conducted by McGlynn et al. [10] focused on examining the effectiveness of hybrid liquid–plasma functionalization in enhancing the stability of carbon nanotubes while suspended in a base fluid. The objective of this research was to explore the potential uses of these stabilized carbon nanotubes in the field of solar energy conversion. The stability of nanofluids has been enhanced using surface treatments, including plasma-induced non-equilibrium electrochemistry. This allows the redispersion of these fluids under simulated circumstances. The enhanced dispersibility results in an increased absorption coefficient and better thermal profile when subjected to solar simulation.

The sorption effectiveness of biochar-mediated ZrFe_2_O_5_ nanocomposites for the treatment of Tartrazine dye-containing textile wastewater was explored by Perveen et al. [11]. Batch mode experimentations were used for this objective. The researchers noted a significant sorption capacity of BC-ZrFe_2_O_5_ NCs for Tartrazine dye. The use of a surface approach has been employed to optimize the parameters of the process.

Kanungo et al. [12] conducted a theoretical investigation of the electrical and electrochemical characteristics of LiMPO_4_ materials, namely those based on iron, cobalt, manganese, vanadium, and chromium. The purpose of their research was to explore the potential of these materials for cathode design in lithium-ion batteries. The researchers used a computational method known as density functional theory, which is based on fundamental principles, to perform their calculations. The participants thoroughly examined the cathode design, including an analysis of several materials and their performances. This analysis specifically focused on key factors such as the Lithium intercalation energy inside the olivine structure, the cohesive energy of the material, and the intrinsic diffusion coefficient across the Lithium channel. The possibility of an open circuit and equilibrium at different charge levels of lithium batteries was also a topic of discussion. It was observed that the choice of metal atom significantly influenced the extent of lithium diffusion inside the olivine structure and the overall energetics of different LiMPO_4_ compounds.

Sharma et al. [13] examined the effects of entropy production, heat transfer, and mass transfer within the context of the Jeffrey fluid model, including solar radiation as a contributing factor. The researchers used a polyvinyl alcohol–water mixture as the primary fluid medium, including copper nanoparticles and microorganisms. The ongoing research aims to create a robust numerical model that accurately represents the flow and thermal properties of a parabolic trough surface collector (PTSC) integrated into a solar plate. This endeavor is driven by the growing use of solar plates in diverse applications. It was shown that increasing the volume fraction of the nanoparticles had a positive impact on the thermal profile. However, the concept of entropy demonstrates a tendency towards increased order because of diffusion factors.
